# Is scientific knowledge socially constructed? A Bayesian account of *Laboratory Life*

**DOI:** 10.3389/frma.2023.1214512

**Published:** 2023-08-02

**Authors:** Henry Small

**Affiliations:** SciTech Strategies Inc., Bala Cynwyd, PA, United States

**Keywords:** scientific knowledge, social construction, Bayes' theorem, *Laboratory Life*, theory and evidence, social factors

## Abstract

In the book *Laboratory Life* Latour and Woolgar present an account of how scientific “facts” are formed through a process of microsocial interactions among individuals and “inscription devices” in the lab initially described as social construction. The process moves through a series of steps during which the details and nature of the object become more and more certain until all qualifications are dropped, and the “fact” emerges as secure scientific knowledge. An alternative to this account is described based on a Bayesian probabilistic framework which arrives at the same end point. The motive force for the constructivist approach appears to involve social processes of convincing colleagues while the Bayesian approach relies on the consistency of theory and evidence as judged by the participants. The role of social processes is discussed in Bayesian terms, the acquisition and asymmetry of information, and its analogy to puzzle solving. Some parallels between the Bayesian and constructivist accounts are noted especially in relation to information theory.

## 1. Introduction

In 1979 Bruno Latour and Steve Woolgar published their iconic book *Laboratory Life* based on Latour's experience as a “participant observer” at the Salk Institute. Chapter 3 of that book is a case study of the “social construction of a scientific fact.” The “fact” in question is the existence and structure of thyrotropin releasing factor, TRF, for which Roger Guillemin and Andrew Schally were awarded the Nobel Prize in 1977. Latour and Woolgar claim to show how this chemical entity was brought into existence and assumed a factual status as the result of the process of “social construction.” The authors assert that its existence is due to social processes independent of its physical existence.

The purpose of this paper is to show that an alternative and more plausible account of the taken-for-granted or “fact” status of TRF is possible based on a probabilistic treatment of hypotheses and evidence in a Bayesian framework. What emerges from this analysis is that the factual status of a biological substance such as TRF or DNA (Small, 2023) is due to its high probability based on the evidence, rather than the operation of social processes. Social and other contingent factors can either accelerate or impede changes in hypotheses or the acquisition of evidence but do not affect the probability that the hypothesis is correct. There is some indication from the text of *Laboratory Life* that such a probabilistic perspective is not that far removed from the fact construction process that the authors endorse. However, the Bayesian and constructivist processes prove to be incompatible.

Our discussion will center on the 1979 edition of *Laboratory Life*. A second edition appeared in 1986 to which a postscript was added but otherwise without major revisions. One notable change was dropping the word “social” from “social construction” which was possibly motivated by Latour's objections to the Strong Program in sociology (Bloor, 1999). We will follow Cole (1992) in regarding Latour and Woolgar's position as a variant of social construction considering the prominent role of microsocial interactions in *Laboratory Life*.

Another excellent source for the Guillemin and Schally pursuit of hormone releasing factors is Nicholas Wade's *The Noble Duel* (1981). Wade's book is a detailed technical history of the search for various releasing factors whereas Latour and Woolgar's book is an extended argument for the “social constructivist” interpretation of this history. Wade, by contrast, focuses more on the competition for priority between Guillemin and Schally taking a more traditional, Mertonian view of scientific activity. In developing a Bayesian account of the TRF story, we rely on the Latour and Woolgar and Wade books, along with historical accounts by Guillemin and Schally themselves. However, the Bayesian account which we offer is an alternative to both the constructivist and Mertonian viewpoints.

## 2. Social construction according to Latour and Woolgar

Latour singled out TRF (thyrotropin releasing factor) for special attention in Chapter 3 of *Laboratory Life* which is entitled “The construction of a fact: the case of TRF(H).” Work on this “hormone releasing factor” was background to the research program of the Salk Institute lab under Roger Guillemin at the time of Latour's stay at the lab from 1975 to 1977 (Latour and Woolgar, 1979, p. 39). The “factual” status of TRF had been achieved prior to Latour's arrival following a multi-year research effort by Guillemin and his competitor Schally culminating in 1969 in a definitive structure for TRF. Latour conducted his case study of TRF using the traditional materials of the historian including the primary published literature, interviews with participants, bibliometric data, and informal materials like lab notes and correspondence. He could not employ the anthropological techniques of participant observation because the decisive work on TRF had been done 6 years prior to his arrival at the Salk Institute and at a different institution, namely Baylor College of Medicine in Houston, Texas and the Veterans Administration Hospital in New Orleans, Louisiana.

Nevertheless, Latour and Woolgar distanced themselves from typical historical treatments which they claim take for granted the factual status and “reality” of the knowledge they are tracing historically. They were interested in the “process by which scientists make sense of their observations” (Latour and Woolgar, 1979, p. 32) which consists of microsocial exchanges occurring in the daily activities of scientists. These exchanges, they claim, result in the construction of scientific “facts.” The authors link fact formation to the dropping of “modalities” (qualifiers) in statements that emerge from the many inscription processes that occur in and outside the lab: “A laboratory is constantly performing operations on statements adding modalities, citing, enhancing, diminishing, borrowing, and proposing new combinations… in situations where a statement is quickly borrowed, used and reused, there quickly comes a stage where it is no longer contested. Amidst the general Brownian agitation, a fact has then been constituted” (Latour and Woolgar, 1979, p. 86). They refer to the collection of microsocial operations as the “agnostic field” perhaps to emphasize its independence from presuppositions about what “facts” might emerge.

In Chapter 4 they describe what happens when a fact becomes established. In addition to the dropping of modalities, the “fact” becomes “stabilized” and takes on a life of its own. Participants forget about the social processes and doubts that preceded its stabilization. The “fact” then becomes part of the reality “out there” even though it is an illusion or fiction constructed within the lab. They state that a “fact” can also be deconstructed, by becoming qualified or doubted, and that these changes can take place day-to-day in microsocial exchanges.

While this formulation is attractive, the question remains: What has motivated the dropping of modalities and the acceptance of some statements as fact? Toward the end of the book the authors introduce a seemingly mechanistic process of narrowing down the alternatives by analogy to the game of Go. How this narrowing occurs is not explained and it appears to introduce an *ad hoc* process in which alternatives are eliminated by logical reasoning. However, as Stephen Cole argues (Cole, 1992, p. 59–81), evidence plays no role in constructivist accounts of how science is done. Latour and Woolgar, along with other social constructivists, fail to show how social processes influence scientific findings, how “facts” could have turned out differently if social conditions had been different, or why the same “facts” can be discovered by different investigators.

The similarity of Latour and Woolgar to our probabilistic interpretation is revealed by the recurrent use of phrases such as “constructing an ordered account out of the apparent chaos of available perceptions,” or how “an ordered account is fabricated from disorder and chaos.” There is a foreshadowing of our approach when, in the context of what they call “inscription devices,” they state that there is a “…tendency to think of inscriptions as confirmations or evidence for or against particular ideas, concepts or theories” (Latour and Woolgar, 1979, p. 63). This tendency is, however, rejected for their anthropological approach because the output of inscription devices, e.g., scientific instruments, would involve the participant observer in the “mythology” of the lab.

In the final chapter entitled “the creation of order out of disorder” there is reference to information theory and the concept of entropy, for example, how “mass spectrometry limited the number of probable statements” on the structure of TRF which had previously been considered equally probable alternatives. In information theory terms, transforming equally probable statements into unequally probable ones reduces the entropy of the lab. An analogy to Maxwell's demon is introduced to decrease entropy in the lab and allow facts to emerge (Latour and Woolgar, 1979, p. 245).

Leon Brillouin's book on information theory is cited (Brillouin, 1962) and its formulation of information as a function of the probability of a statement. The more unexpected the statement, the greater its information content. The objective of the microsocial maneuvers, they claim, is to convince others that not all statements are equally probable. This is seen as important for moving statements toward fact-like status. The authors also regard mass spectroscopy as having higher credibility than chromatography, implying a higher information content and higher probability. Likewise, the cost of objecting to the findings from mass spectrometry by suggesting alternative explanations becomes prohibitive. Established science is, then, a set of statements that is too costly to modify due to their certainty. The approach of the authors is summarized by the quote: “Scientific activity is not about nature, it is a fierce fight to construct reality” (Latour and Woolgar, 1979, p. 243).

In what follows, we will present an alternative interpretation of TRF history in which ideas from information theory, probability, and entropy emerge in a natural way from a Bayesian approach.

## 3. Bayesian approach to theory and evidence

In contrast to the Latour and Woolgar approach to establishing “facts,” the Bayesian approach views the history of TRF as a sequence of models put forward at various times along with evidence for or against them. We will refer to these models interchangeably as theories or hypotheses. The models are also evaluated against alternative or competing models using the same evidence. Models, hypotheses, or theories in the Bayesian framework are generalizations which purport to be true about which we have varying degrees of confidence. Evidence is time specific and accepted to be true, at least to some degree, and is usually observational or experimental in nature. In complex cases, theories and evidence can be linked together in directed, acyclic graphs called Bayesian networks (Koller and Friedman, 2009, p. 62) where the arrows stand for dependencies of a probabilistic nature.

Our initial degree of confidence in a theory before looking at the evidence is its prior probability expressed as P(T). Theory and evidence can cohere or not cohere to varying degrees if, for example, a consequence of a theory agrees or disagrees with an experimental result, for example, by deriving it from theory. If the evidence is consistent with the theory, then by Bayes' theorem the probability of the theory given the evidence increases by induction unless an alternative theory is more consistent with the same evidence. Alternative theories can be either explicit or implicit.

In assessing theories against evidence what needs to be determined is their degree of consistency or compatibility. That is, how securely is the evidence a consequence of the theory? This is expressed as a conditional probability of the evidence given the hypothesis or theory, expressed as P(E|T) which is called a likelihood. For example, in the history of hormone releasing factors, the anatomical fact that portal blood vessels connect the hypothalamus to the anterior pituitary was weakly consistent with the idea that some substance was being transmitted from one location to the other. However, this was circumstantial evidence and only weakly consistent. Subsequently it was also shown experimentally that cutting these vessels prevented the pituitary from releasing a hormone required for sexual reproduction, and this provided stronger evidence at a higher probability or likelihood.

For historical reconstructions, the procedure is to estimate these probabilities from the point of view of the historical participants. For example, evidence generated by experiment or direct observation usually carries more weight than evidence based on rules of thumb or analogy to other systems. As shown in a previous study (Small, 2023), the probabilities need only be specified approximately using a preset scale ranging qualitatively from “strongly consistent” to “strongly inconsistent” with 0.5 defined as neutral (Table 1). The neutral state means that there are even odds that a piece of evidence is consistent or inconsistent with some hypothesis. The conditional probabilities range from 0.3 to 0.7, avoiding extremes of 0 or 1 (absolute uncertainty or certainty) which do not seem realistic in historical contexts where uncertainty is generally high. For example, according to this scheme the weakly consistent evidence offered by the existence of portal blood vessels is assigned a conditional probability of 0.6 but the evidence offered by experimentally blocking the vessels is given a 0.7 probability. P(E|T) is the probability that given the theory is correct, the evidence will be seen.

**Table 1:** Conditional probability scale for fit between theory and evidence.

**P(E|T)**	**Code**	**Description**
0.7	SC	Strongly consistent
0.6	WC	Weakly consistent
0.5	N	Neutral
0.4	WI	Weakly inconsistent
0.3	SI	Strongly inconsistent

The conditional probability of some evidence given a theory is qualitatively assessed from the historical record and assigned an approximate value ranging from 0.3 for “strongly inconsistent” theory and evidence to 0.7 for “strongly consistent.

When multiple lines of evidence are taken into account and those different types of evidence are conditionally independent (Koller and Friedman, 2009, p. 24), the form of Bayes' theorem is given by the formula (1) below where P(T|E_i, n_) is the posterior probability of the theory given *n* forms of evidence, P(T) is the prior probability of theory T, and P(E_i_|T) are the likelihoods for the various forms of evidence i. P(~T), the probability of “not T,” is the probability of the alternative hypotheses. P(~T) equals 1 – P(T) because the set of all possible theories must sum to 1. If the posterior is greater than the prior, the hypothesis T is regarded as confirmed and disconfirmed if it is less than the prior.


(1) $$P\left(T|E_{i},n\right)=\frac{P\left(T\right)\prod_{i}P\left(E_{i}|T\right)}{P\left(T\right)\prod_{i}P\left(E_{i}|T\right)+P\left(\sim T\right)\prod_{i}P\left(E_{i}|\sim T\right)}$$

This formulation is sometimes referred to as naïve Bayes and can be represented as a network with a central node standing for the theory from which arrows point outward to the various forms of evidence explained or predicted by the theory. In this paper we will consider each of the various models of the TRF substance as theories or hypotheses. The most strongly confirmed model of TRF, as indicated by the highest posterior probability, will correspond to what Latour and Woolgar refer to as the constructed “fact.” In other words, what they call a “fact,” we call a model having high probability.

The symbol π in the formula (1) indicates that the likelihoods for each form of evidence are multiplied together. It is possible by simple algebra to show that this formulation is equivalent to each line of evidence considered individually provided the posterior of applying one form of evidence is used as the prior for the next form. The numerator is the product of the likelihoods and the prior for the theory being tested and expresses how well the theory fits the evidence. The first term in the dominator is the same as the numerator and the second term is the product of the likelihoods for the alternative theories times the prior probability of the alternative theories, 1 minus the prior. This second term expresses how well the alternative theories fit the evidence. If the posterior equals the prior, the theory is not confirmed or disconfirmed which occurs, for example, if the product of likelihoods of T equals the likelihoods of ~T. Setting P(T) and P(~T) to 0.5 is an example of uniform priors and corresponds to the state of the highest uncertainty and maximum entropy as will be shown later. This is sometimes referred to as the “principle of indifference” (Howson, 2008).

We will present each model or theory as a table with the prior probabilities, P(T) and P(~T), given at the top of the table followed by the conditional probabilities for each form of evidence with respect to both T and ~T (the likelihoods), and the computed posterior at the bottom of the table. The posterior can then be computed using the formula (1) above from the data in the table. For each new model or theory, the prior probability is set to the posterior probability of the previous model in the series provided the object of inquiry, or what the model is supposed to explain, has not changed. This can be interpreted psychologically as meaning that the participants confidence in the new hypothesis is dependent on their previous experience with other models.

## 4. Bayesian history of releasing factors

Latour and Woolgar divide the releasing factor story into two parts. The first part was establishing that “releasing factors” exist in the hypothalamus which control the secretion of various hormones by the pituitary. The chief proponent of this idea was the British physiologist Geoffrey Harris whose 1955 book was entitled “Neural control of the pituitary gland.” This early work was carried out primarily on a proposed factor CRF (corticotropin releasing factor) which was thought to cause the pituitary to release ACTH (corticotropin). ACTH in turn stimulated the adrenal glands to secrete cortisone. Hans Selye considered ACTH part of the stress response and called the hypothetical CRF the “first mediator” because it initiated the response. The idea that the hypothalamus released different “factors” for each pituitary hormone was countered by the physiologist Samuel McCann at the University of Pennsylvania who thought that the hormone vasopressin (or oxytocin) from the posterior pituitary controlled the release of ACTH (Wade, 1981, p. 71).

The second part of the releasing factor story was to figure out the chemical composition for one of the factors. By about 1960, work had shifted away from the CRF-ACTH system to the TRF-TSH system (thyrotropin releasing factor—thyroid stimulating hormone) due to the difficulties encountered in purifying enough CRF (Schally, 1977; Wade, 1981, p. 68).

The quest for the chemical structure of TRF went through several phases. The first phase, up to about 1965, dealt with circumstantial evidence that the factor was some kind of peptide. The second phase, covering the years 1965 to 1968, focused on the idea that the factor was a simple peptide consisting of three amino acids. This claim was eventually rejected, and as shown below, leads to Bayesian disconfirmation. The third phase, which occurred during the single year, 1969, was that TRF is a modified peptide that was somehow blocked or protected at both of its ends. This phase consisted of three steps: first postulating that TRF was blocked at one of its terminals by an acetyl group, second that it was blocked instead by a pyro ring, and third that it was also modified at its other end by an amide group. The reasoning that led to this final form can be interpreted as a Bayesian process leading to a high posterior probability for the structure which accords with Latour and Woolgar's conclusion that a scientific fact had been constructed.

Beginning with the first question, whether releasing factors from the hypothalamus exist, Table 2 summarizes the main lines of evidence. The alternative hypothesis promoted by McCann, was that the hormones vasopressin or oxytocin from the posterior pituitary controlled the pituitary (Latour and Woolgar, 1979, p. 117).

**Table 2:** Evidence for the existence of releasing factors.

T = the theory that releasing factors come from the hypothalamus to the anterior pituitary (Harris)			
~T = the theory of vasopressin or oxytocin controlling the release of hormones from the pituitary (McCann)			
E1 = direction of blood flow thru portal vessels is from the hypothalamus to the pituitary			
E2 = cutting portal vessels stops hormone secretion by the pituitary (Harris)			
E3 = vasopressin can stimulate ACTH release (McCann)			
E4 = hypothalamic fragments introduced to pituitary cell cultures release ACTH (Guillemin and Schally)			
E5 = nerve fibers connecting the hypothalamus to the posterior pituitary contain vasopressin			
**Probability**	**Value**	**Code**	**Description**
P(T)=	0.5		Hypothalamus secretes factors that stimulate the pituitary to secrete various hormones
P(~T)=	0.5		1 – P(T) vasopressin or oxytocin in the posterior pituitary control the release of hormones by the pituitary
P(E1|T)=	0.6	WC	Blood vessels connecting the hypothalamus to the pituitary is **weakly consistent** with transmission of releasing factors
P(E1|~T)=	0.5	N	Blood vessels going to the anterior pituitary is **neutral** to the source of vasopressin from the posterior pituitary
P(E2|T)=	0.7	SC	Cutting portal vessels is **strongly consistent** with blocking the releasing factors from entering the anterior posterior
P(E2|~T)=	0.4	WI	Cutting the vessels to stop ACTH is **weakly inconsistent** with vasopressin controlling ACTH from the posterior pituitary
P(E3|T)=	0.3	SI	Vasopressin stimulation of ACTH is **strongly inconsistent** with a factor from the hypothalamus
P(E3|~T)=	0.7	SC	Vasopressin control of the pituitary is **strongly consistent** with ACTH release
P(E4|T)=	0.7	SC	Hypothalamus tissue in a pituitary cell culture producing ACTH is **strongly consistent** with hypothalamic origin of factors
P(E4|~T)=	0.3	SI	Cell culture release of ACTH is **strongly inconsistent** with vasopressin control of ACTH
P(E5|T)=	0.5	N	Nerve fibers to the posterior pituitary are **neutral** regarding the hypothalamic origin of releasing factors
P(E5|~T)=	0.6	WC	Nerves containing vasopressin connecting to the pituitary is **weakly consistent** with control of ACTH release
P(T|E1-E5)=	0.64	Confirm	

T stands for the theory or model under consideration and ~T (not T) the alternative theory or theories. Lines of evidence are numbered E1 through E5. A brief statement of each theory and line of evidence is given at the top of the table. In the body of the table prior and posterior probabilities are shaded in yellow. Conditional probabilities are shaded green for the theory being tested and pink for the alternative. The column headed Description is a short explanation of the consistency or inconsistency of the theory and evidence, while the Code and Value columns give probabilities from Table 1. The last line in the table gives the posterior probability according to Bayes' rule which confirms if it is greater than the prior. Bolded words in the Description column indicate the relationship between the theory and evidence in accord with the abbreviations in the Code column.

The prior probability P(T) is set to 0.5 to reflect the initial uncertainty regarding the theory prior to the evidence being considered. This automatically fixes the value of the alternative hypothesis to 1 – P(T) = 0.5 since the sum of all hypotheses cannot exceed 1. The conditional probabilities show the consistency or inconsistency of each line of evidence with both the target and alternative theory.

In this early period up to about 1955, the evidence for hormone releasing factors from the hypothalamus was modest and somewhat contradictory. The evidence in Table 2 labeled E1 was an anatomical observation that a system of portal blood vessels connected the hypothalamus to the pituitary. Experiments by Harris cutting or blocking these blood vessels so they could not reconnect with the hypothalamus (E2) were more definitive, as were independent experiments by Guillemin and Schally around 1954 (E4) that anterior pituitary cell cultures to which hypothalamic fragments were added produced ACTH (Schally, 1977; Guillemin and Lemke, 2013). However, McCann (E3) demonstrated that vasopressin could stimulate the release of ACTH independent of the hypothalamus. Despite the mixed evidence, the hypothalamic factor theory was weakly confirmed, encouraging investigators to take the next step toward the isolation and characterization of the factors. Among them were Roger Guillemin and Andrew Schally who had been active in the study of CRF. As Guillemin stated in an interview “the stage was set” for going after this first mediator (Guillemin and Lemke, 2013).

In 1956 Andrew Schally took a position with Guillemin at Baylor College of Medicine in Houston where they worked together on CRF. After 5 years and no clear results, Schally decided to move on and accepted a position at the Veterans Administration Hospital and Tulane University School of Medicine in New Orleans, Louisiana where he could set up his own lab. In 1960 Guillemin had taken on an additional appointment at College de France in Paris and divided his time between Houston and Paris. He decided to set the CRF problem aside and look for other postulated releasing factors such as LRF (luteinizing releasing factor) and TRF (thyrotropin releasing factor). He located a slaughterhouse in France that could provide sheep hypothalamic tissue and used that material to pursue those other factors. Schally, on the other hand, decided to switch to another species for hypothalamic tissue, namely the pig, and arranged with Oscar Mayer and Company to provide the tissues (Schally, 1977; Wade, 1981, p. 94).

An important development in focusing attention on TRF was Guillemin's development of an assay for TSH (thyroid stimulating hormone) which was the hormone released by the pituitary supposedly in response to TRF. The assay involves injecting the thyroid of rats with radioactive iodine which is incorporated into the animal's thyroid hormones. The rats can then be injected with a TRF-like test substance, and the presence of radioactive TSH in their blood indicated if the test material was acting as real TRF. A Geiger counter measured the amount of radioactivity in the blood samples and indicated the purity of the TRF test sample (Wade, 1981, p. 149). Armed with the assay and a variety of chemical separation techniques from chromatography, the work of isolating and characterizing TRF could begin. As in the case of CRF, the TRF search was hampered by its extremely low concentration in hypothalamic tissue, and great quantities of these tissues from sheep or pig would eventually have to be collected, processed, and purified by the various separation methods.

### 4.1. Initial clues that releasing factors are peptides

In Table 3 we present preliminary clues prior to 1964 that TRF and other releasing factors were peptides, that is, chains of amino acids. No alternative hypotheses were apparently considered so the alternative is simply that releasing factors are not peptides. The question of the chemical composition of releasing factors, and specifically TRF, is independent of the question of whether the releasing factors come from the hypothalamus, and we cannot simply use the posterior from Table 2 as the prior for Table 3.

**Table 3:** Initial clues on releasing factors as peptides.

T = releasing factors are peptides			
~T = alternative theory (releasing factors are not peptides)			
E1 = oxytocin and vasopressin found by Vincent du Vigneaud from the posterior pituitary are peptides (1960)			
E2 = glands of ectodermic embryonic origin (e.g., the hypothalamus) synthesize proteins or peptides (David Thompson)			
E3 = a melanophore stimulating peptide was isolated from extracts of the posterior pituitary (Harris, 1955)			
**Probability**	**Value**	**Code**	**Description**
P(T)=	0.5		Even odds that releasing factors are peptides
P(~T)=	0.5		1—prior
P(E1|T)=	0.6	WC	Peptide releasing factors **weakly consistent** with peptides oxytocin and vasopressin and other pituitary hormones
P(E1|~T)=	0.5	N	Alternative theory **neutral** with respect to peptides oxytocin and vasopressin
P(E2|T)=	0.6	WC	Peptide structure of releasing factors **weakly consistent** with embryonic development of ectoderm synthesizing peptides
P(E2|~T)=	0.5	N	Alternative theory **neutral** with respect to ectodermic origin
P(E3|T)=	0.6	WC	Peptide releasing factors **weakly consistent** with melanophore stimulating peptide isolated from the posterior pituitary
P(E3|~T)=	0.5	N	Alternative theory **neutral** with respect to melanophore stimulating peptide
P(T|E1-E3)=	0.63	Confirm	

In the body of the table prior and posterior probabilities are shaded in yellow. Conditional probabilities are shaded green for the theory being tested and pink for the alternative. Bolded words in the Description column indicate the relationship between the theory and evidence in accord with the abbreviations in the Code column.

Based on the principle of indifference, as in the case of the existence of releasing factors, we assign a prior probability of 0.5 (even odds) that releasing factors are peptides making the alternative hypothesis (factors are not peptides) also 0.5. The three lines of evidence are all indirect and weakly consistent. E1 is based on an analogy to the peptide structures of vasopressin and oxytocin from the posterior pituitary discovered by Vincent du Vigneaud (Hofmann, 1987) for which, in part, he had received a Nobel Prize in 1955. The second clue (E2) was provided by the embryological observation that glands of ectodermic origin synthesize peptides (Guillemin and Lemke, 2013). The third form of evidence (E3), again an analogy, was that a melanophore stimulating peptide had been isolated from the pituitary. The conditional probabilities for the alternative non-peptide nature of releasing factors are all coded as neutral since it is not known how a “non-peptide” theory would relate to the evidence. At this early stage the evidence pointed to a weak confirmation of the peptide nature of releasing factors.

### 4.2. TRF as a simple peptide

As research efforts focused on TRF, evidence accumulated that both supported and contradicted TRF's peptide make up. At the same time purification methods advanced, for example, using Sephadex columns for separation and the development of other chromatographic methods (Schally, 1977). The fractions resulting from these separations could be subject to the TSH assay as well as amino acid analysis using acid hydrolysis. Table 4 summarizes the evidence from 1964 up to 1968 that TRF was a simple peptide consisting of three amino acids. Here we use the posterior from Table 3 as the prior for Table 4 because the objective had not changed. The alternative hypothesis is again that TRF is not a peptide.

**Table 4:** TRF as a simple peptide.

T = TRF consists of the three peptides glu, his, and pro			
~T = TRF consists mainly of non-peptide material			
E1 = chemical tests showed TRF contains three amino acids: glu, his, and pro in equal amounts			
E2 = only a fraction (8–30%) of the purified TRF material was found to consist of peptides			
E3 = proteases were not able to completely degrade the purified material			
E4 = synthetic combinations of the three peptides did not show TRF activity			
**Probability**	**Value**	**Code**	**Description**
P(T)=	0.63		Posterior of initial clues on structure (from Table 3)
P(~T)=	0.37		1—prior
P(E1|T)=	0.7	SC	Peptide chemistry is **strongly consistent** with TRF being a simple peptide consisting of 3 amino acids glu, his and pro
P(E1|~T)=	0.4	WI	The presence of peptides in TRF is **weakly inconsistent** with the non-peptidic nature of TRF
P(E2|T)=	0.3	SI	A majority of TRF being non-peptidic by weight is **strongly inconsistent** with its being a simple peptide
P(E2|~T)=	0.7	SC	The dominance of the non-peptide portion of TRF is **strongly consistent** with its not being a simple peptide
P(E3|T)=	0.3	SI	The failure of protease enzymes to completely degrade TRF is **strongly inconsistent** with its being a simple peptide
P(E3|~T)=	0.7	SC	The failure to completely degrade TRF is **strongly consistent** with its non-peptide nature
P(E4|T)=	0.3	SI	The absence of activity of synthetic combinations of the three amino is **strongly inconsistent** with TRF as a simple peptide
P(E4|~T)=	0.7	SC	The non-peptide nature of TRF is **strongly consistent** with the inactivity of combinations of three amino acids
P(T|E1-E4)=	0.19	Disconfirm

In the body of the table prior and posterior probabilities are shaded in yellow. Conditional probabilities are shaded green for the theory being tested and pink for the alternative. Bolded words in the Description column indicate the relationship between the theory and evidence in accord with the abbreviations in the Code column.

While it was possible to detect the amino acids glutamic acid, histidine, and proline (abbreviated as glu, his, and pro), three of the four lines of evidence pointed to the non-peptide nature of TRF and a strong disconfirmation of the simple peptide hypothesis.

In 1964 Guillemin, together with a chemist Darrell Ward at nearby M. D. Anderson Hospital, had concluded that TRF was a peptide consisting of 11 amino acid units. In 1965 this was revised to 18 units consisting of his, glu and pro (E1) whose order was unknown. In 1965 Schally found TRF to consist of 23 units of the same three amino acids. However, the peptide portion of the TRF molecule comprised only 8 percent by weight of the purified material in Guillemin and Ward's experiments, while in Schally's case, the amino acids accounted for 30 percent (E2). Thus, most of the purified material was non-peptidic in nature for both investigators. In addition, if TRF were a peptide it would be degraded by proteolytic enzymes, but this was not the case. Some biological activity was still observed after treatment with proteases (E3). Finally, Schally asked colleagues at Merck to synthesize all six permutations of the three amino acids (his, glu, and pro), but none of the permutations showed any biological activity (E4). By 1966 both teams had declared that TRF was not a simple peptide (Latour and Woolgar, 1979, p. 129; Wade, 1981, p. 113–116). Thus, Bayesian disconfirmation is consistent with the statements of Guillemin and Schally prior to 1968.

### 4.3. TRF as a modified peptide

In late 1968 Roger Burgus, a chemist working with Guillemin, obtained new results indicating that 80% by weight of the most highly purified TRF consisted of only the three amino acids in close to equimolar amounts. What the remaining 20% consisted of he did not know, but it was possible that it was due to remaining impurities (Wade, 1981, p. 145). The amino acids were the same three that had been detected earlier by the two groups. Burgus' findings were presented at a symposium in Tucson, Arizona in January 1969. The symposium was convened by the NIH study section on endocrinology that had been supporting the work on releasing factors by Guillemin, Schally, and others. NIH was concerned that insufficient progress had been made after many years of investment and the study section was considering whether it was worthwhile to continue funding the effort (Latour and Woolgar, 1979, p. 139; Wade, 1981, p. 136). Burgus' new results had come in the nick of time, 3 weeks before the Tucson meeting. Equally important as the new 80% number was Burgus' finding that the N-terminal of the TRF peptide was not “free,” that is, was not occupied by the usual NH_2_ group found in other amino acids. Burgus reasoned that if the N-terminal was missing, it must be “blocked” or “protected” by some other chemical group. This was known to be the case for some natural peptides whose ends were blocked by acetyl groups.

During the meeting Guillemin put a call through to an acquaintance at Hoffmann-La-Roche in Switzerland to see if they could synthesize the six permutations of three amino acids (glu, his, and pro) as Schally had done earlier with Merck. Six weeks later Burgus determined, as Schally had earlier, that none of the six showed TRF activity. However, he then acetylated each form and found to his surprise that only one of the permutations, acetyl-glu-his-pro, showed activity. According to Wade this was the most thrilling moment of Burgus' career. However, the activity of the acetylated tripeptide was less than the activity of the purified natural material (Wade, 1981, p. 150).

Table 5 summarizes the evidence for and against the idea that TRF was an acetylated peptide. The alternative hypothesis is that TRF was a simple peptide, using the posterior from Table 4 as the prior for Table 5. The absence of a free NH_2_ at the N-terminal was consistent with the presence of another chemical entity such as an acetyl group (E1). This idea is also consistent by analogy with other natural peptides having this structure (E2). The unexpected activity of one of the acetylated sequences (acetyl-glu-his-pro) was consistent with acetyl-glu-his-pro being similar to actual TRF (E3). Finally, the acetyl hypothesis is inconsistent with its lower biological activity than purified TRF (E4). Nevertheless, taking all the evidence into consideration, the hypothesis is confirmed, a reversal from the disconfirmation of the previous simple tripeptide model in Table 4. The Bayesian reversal is also reflected in Burgus' reaction to his surprising finding of activity by only one of the possible amino acid permutations.

**Table 5:** TRF as an acetylated peptide.

T = the TRF peptide is blocked at the N-terminal by an acetyl group			
~T = TRF consists only of the three amino acids: glu, his, and pro			
E1 = no NH_2_ group was found at the N-terminal of the TRF peptide			
E2 = in some natural peptides the N-terminal is blocked by an acetyl group (analogy)			
E3 = one of the six acetylated combinations of the three amino acids was active using the TRF assay			
E4 = the acetylated form was not as active as purified natural TRF			
**Probability**	**Value**	**Code**	**Description**
P(T)=	0.19		Posterior from simple peptide glu-his-pro
P(~T)=	0.81		1—prior
P(E1|T)=	0.7	SC	The absence of an NH_2_ group on the N-terminal is **strongly consistent** with an acetyl group at the N-terminal
P(E1|~T)=	0.3	SI	The absence of an NH_2_ group on the N-terminal is **strongly inconsistent** with TRF as a simple peptide
P(E2|T)=	0.6	WC	An acetyl group on the N-terminal of TRF is **weakly consistent** with other natural peptides
P(E2|~T)=	0.4	WI	An acetyl group on the N-terminal of a simple peptide is **weakly inconsistent** with its being a simple peptide
P(E3|T)=	0.7	SC	The TRF activity of acetylated glu-his-pro is **strongly consistent** with TRF having that sequence
P(E3|~T)=	0.3	SI	The absence of activity of the glu-his-pro sequence without acetylation is **strongly inconsistent** with TRF as a simple peptide
P(E4|T)=	0.4	WI	The lower activity of acetylated glu-his-pro is **weakly inconsistent** with it being identical to natural TRF
P(E4|~T)=	0.5	N	The lower activity of the acetylated form is **neutral** to TRF as a simple peptide
P(T|E1-E4)=	0.61	Confirm	

In the body of the table prior and posterior probabilities are shaded in yellow. Conditional probabilities are shaded green for the theory being tested and pink for the alternative. Bolded words in the Description column indicate the relationship between the theory and evidence in accord with the abbreviations in the Code column.

The ironic twist is that Burgus was only partly correct. The activity he thought was due to acetyl-glu-his-pro was a chimera. Back in Houston, Burgus heard from chemists at Hoffmann-La Roche who had provided the tripeptides that glu-his-pro might have formed an internal ring at the N-terminal during acetylation. The ring was called pyro-glutamic acid or pyro-glu and it was known that several natural proteins had pyro-glu terminals (Wade, 1981, p. 154). Guillemin and Burgus then asked the Swiss chemists to synthesize samples of pure acetyl-glu-hist-pro and pyro-glu-hist-pro. It turned out that pyro-glu-hist-pro was active while the acetyl form was not using the TRF assay. But its activity was still less than that of their purified natural TRF. While they had made headway, questions remained. Guillemin quoted himself as saying: “This is still not the final molecule” (Guillemin and Lemke, 2013).

Table 6 summarizes the evidence regarding the pyro-glu-his-pro hypothesis. The pyro-glu ring was consistent with the acetylation process (E1). That pyro-glu-his-pro was biologically active, and acetyl-glu-his-pro was inactive, reversing Burgus' earlier conclusion, was consistent with TRF as having the pyro form (E2). The fact that the synthetic pyro form was still not as active as natural TRF was inconsistent with regarding it as real TRF (E3). The posterior of 0.84 suggests that they were closer to an answer and perhaps on the right track.

**Table 6:** TRF has a pyro ring at its N-terminal.

T = TRF peptide consists of glu-his-pro blocked at the N-terminal by a pyro ring			
~T = TRF consists of glu-his-pro blocked by an acetyl group at the N-terminal			
E1 = glu-his-pro was known to form an internal ring at the N-terminal during acetylation			
E2 = synthetic pyro-glu-his-pro was active in the assay while the acetyl form was not			
E3 = the pyro and acetyl forms are not as active as the purified TRF in the assay			
**Probability**	**Value**	**Code**	**Description**
P(T)=	0.61		Posterior of acetyl-glu-his-pro
P(~T)=	0.39		1—prior
P(E1|T)=	0.6	WC	A pyro ring at the N-terminal is **weakly consistent** with the acetylation of glu-his-pro
P(E1|~T)=	0.4	WI	Acetyl-glu-his-pro is **weakly inconsistent** with acetylation forming a pyro ring
P(E2|T)=	0.7	SC	TRF as pyro-glu-his-pro is **strongly consistent** with its TRF activity
P(E2|~T)=	0.3	SI	TRF as acetyl-glu-his-pro is **strongly inconsistent** with its absence of TRF activity
P(E3|T)=	0.4	WI	TRF as pyro-glu-his-pro is **weakly inconsistent** with its lower TRF activity than natural TRF
P(E3|~T)=	0.4	WI	TRF as acetyl-glu-his-pro is **weakly inconsistent** with its lower TRF activity than natural TRF
P(T|E1-E3)=	0.84	Confirm

In the body of the table prior and posterior probabilities are shaded in yellow. Conditional probabilities are shaded green for the theory being tested and pink for the alternative. Bolded words in the Description column indicate the relationship between the theory and evidence in accord with the abbreviations in the Code column.

According to Guillemin it was Burgus who proposed, “Maybe the C terminus is also blocked” (Guillemin and Lemke, 2013). Exactly what prompted Burgus to think that it was blocked by an amide group (CONH_2_) is not clear. Once again analogy may have played a role because in an article published in June, 1969 they stated “…there exist a number of biologically active polypeptides of which the terminal amino acid has been amidated” (Burgus et al., 1969a; Wade, 1981, p. 156). Although infrared spectroscopic and nuclear magnetic resonance tests showed pyro-glu-his-pro-amide to be very similar to TRF, the findings were inconclusive, and Guillemin did not initially accept the amide form as equivalent to TRF perhaps because it would not provide the molecular weight consistent with the missing 20% non-peptide material.

Meanwhile Schally's group had expanded to include chemists Karl Folkers and his postgraduate fellow Franz Enzmann at the University of Texas, Austin. Folkers had attended the Tucson meeting and was eager to work on the TRF problem. Schally provided Folkers with a sample of his purified TRF and the synthetic tripeptides from Merck. Enzmann went to work trying to find the missing non-peptide portion of TRF which Schally had said was as high as 70%, not accepting the new 20% finding by Burgus. Working with the tripeptide glu-his-pro, he “blocked” the carboxyl groups by what was considered the standard procedure which was to amidate them. He thereby accidentally created, by an unintended side reaction, glu-his-pro-amide, among other products. Schally's group then carried out the TRF assay on the new synthetic products and found one had activity. Schally phoned Enzmann and said “We've got it” (Wade, 1981, p. 158). However, Schally did not accept that Enzmann's reactions had produced TRF because the molecular weight of the non-peptide portion was below the 70% he expected and the product still had, he thought, an unblocked N-terminal. Enzmann resolved that issue by searching the chemical literature: the amidation process he performed was known to cause the glutamic acid end to form cyclic pyro-glu at the N-terminal. Thus, one unintended byproduct of his reactions was actually pyro-glu-his-pro-amide and it had a blocked N-terminal as pyro-glu and was not “free” as Schally had feared. However, Schally was still not convinced that he had found the correct form for TRF.

At this point in June 1969 both Guillemin and Schally's groups had both hit upon pyro-glu-his-pro-amide and had seen its close similarity to purified TRF in various tests and assays (Schally, 1977). The breakthrough for Guillemin came in the fall of 1969 when Burgus finally succeeded in obtaining mass spectra for purified TRF and synthetic pyro-glu-his-pro-amide and showed that they produced identical fragments (Guillemin, 1977; Latour and Woolgar, 1979, p. 148; Wade, 1981, p. 167). For Guillemin this was conclusive proof of their equivalence, and he was triumphant. He quickly wrote up the finding for the French journal *Comptes Rendus* which was published on November 12th (Burgus et al., 1969b). However, another Folkers-Schally paper had scooped them by 6 days. On November 6, an article appeared in *Biochemical and Biophysical Research Communications* stating that the structure of TRF was pyro-glu-hist-pro-amide. In it they showed that TRF and pyro-glu-hist-pro-amide could not be distinguished in 17 different chromatographic systems and by the TRF bioassay (Boler et al., 1969). Nevertheless, Guillemin claimed victory stating that only his group had shown decisively that TRF was identical to pyro-glu-hist-pro-amide using mass spectroscopy, and that thus his group deserved priority for the discovery. The Guillemin team held a party to celebrate. Schally's reaction was more subdued because he had to share the glory with the latecomer Folkers. In 1970 Schally confirmed the Guillemin team's mass spectroscopy results (Wade, 1981, p. 177).

Table 7 shows four lines of evidence confirming TRF as pyro-glu-his-pro-amide. Enzmann's work on amidation was consistent with the amide form because it showed how a pyro ring could form which led to TRF activity (E1). The analogy of an amide form of TRF was consistent with the chemical structure of other biologically active peptides (E2). Burgus' mass spectroscopic results were strongly consistent with the molecular fragments expected from the amide form (E3). Finally, the indistinguishability of natural TRF from the synthetic amide form across multiple chromatographic tests and assays by the Folkers-Schally team was also strongly consistent with TRF having the amide form (E4). The posterior probability of 0.99 is consistent with Guillemin team's exuberance, and the emergence of a “fact.”

**Table 7:** TRF as pyro-glu-his-pro amide.

T = the TRF peptide is protected at both the N- and C-terminal ends as pyro-glu-his-pro-amide			
~T = TRF is pyro-glu-his-pro			
E1 = when the glutamic acid end is amidated it has the tendency to form the internal ring known as pyro-glu			
E2 = the amide form of pyro-glu-his-pro is analogous to other biologically active polypeptides			
E3 = a comparison of purified TRF vs. synthetic pyro-glu-hist-pro-amide by mass spectroscopy showed them to be identical			
E4 = purified TRF and synthetic pyro-glu-his-pro-amide could not be distinguished in 17 different chromatographic systems and assays			
**Probability**	**Value**	**Code**	**Description**
P(T)=	0.84		Posterior of pyro-glu-his-pro
P(~T)=	0.16		1—prior
P(E1|T)=	0.6	WC	Pyro-glu-his-pro-amide is **weakly consistent** with amidation
P(E1|~T)=	0.4	WI	Amidation is **weakly inconsistent** with TRF as pyro-glu-his-pro
P(E2|T)=	0.6	WC	The amide form of TRF is **weakly consistent** with other biologically active peptides
P(E2|~T)=	0.4	WI	TRF as pyro-glu-his-pro is **weakly inconsistent** with other biologically active peptides
P(E3|T)=	0.7	SC	Mass spectroscopy of TRF showed fragments **strongly consistent** with pyro-glu-his-pro-amide
P(E3|~T)=	0.3	SI	Mass spectroscopy of TRF showed fragments **strongly inconsistent** with pyro-glu-his-pro
P(E4|T)=	0.7	SC	The indistinguishability of pyro-glu-his-pro-amide from natural TRF across chromatographic tests is **strongly consistent** with their equivalence
P(E4|~T)=	0.3	SI	The ability of pyro-glu-his-pro to be distinguished from natural TRF by chromatographic tests is **strongly inconsistent** with their equivalence
P(T|E1-E4)=	0.99	Confirm	

In the body of the table prior and posterior probabilities are shaded in yellow. Conditional probabilities are shaded green for the theory being tested and pink for the alternative. Bolded words in the Description column indicate the relationship between the theory and evidence in accord with the abbreviations in the Code column.

**Figure 1:**
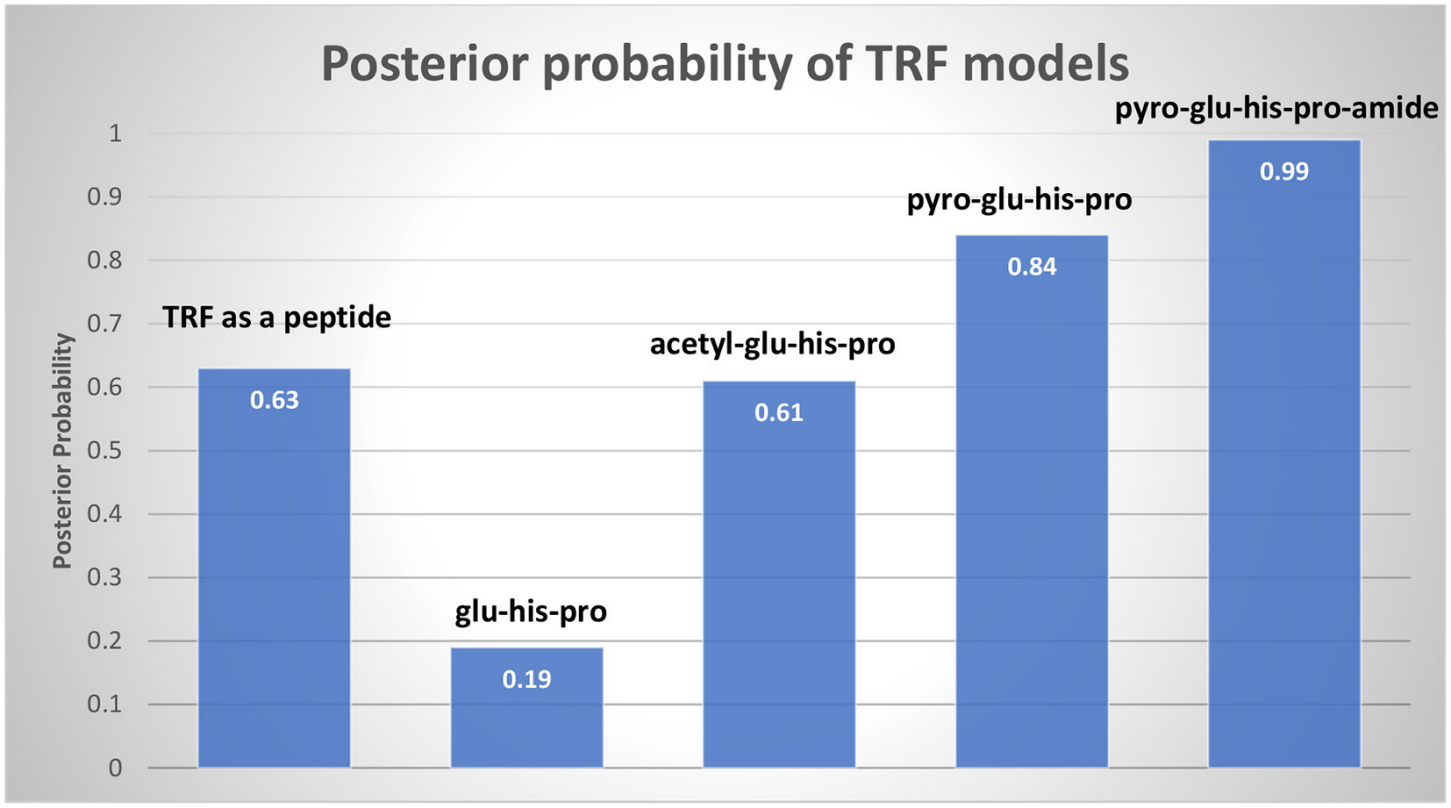
The posterior probabilities of the five TRF models are presented in chronological order from left to right. Since the prior of each model was set to the posterior of the previous model, the second model, glu-his-pro, was disconfirmed, its posterior being less than its prior. The three subsequent models are however confirmed.

Figure 1 shows the change in posterior probability across the five main models: peptide, glu-his-pro tripeptide, acetyl-glu-his-pro, pyro-glu-his-pro, and pyro-glu-his-pro-amide. Since each subsequent model takes the posterior of the previous model as its prior, a decrease in the posterior indicates a disconfirmation, as in the simple peptide model containing three amino acids. An increase in the posterior indicates confirmation, and the ascending pattern for the last three models, all proposed during 1969, signals what Lakatos would call a “progressive problem shift” (Lakatos, 1970) where each new model explains additional empirical findings. The discovery was less dramatic and surprising than, for example, the structure of DNA by Watson and Crick (Small, 2023), which saw an abrupt increase in the posterior. In the TRF case, the largest increase occurred going from the simple tripeptide model to the acetyl-glu-his-pro model which Burgus experienced as a thrilling moment.

## 5. The role of social factors

In what sense was TRF socially constructed? As we have shown, a Bayesian confirmation process supposedly reaches the same end point as Latour and Woolgar's social construction: the formation of new and certain knowledge. Our process was driven by the fit, what we have called the “consistency” of theoretical claims (models of TRF) with diverse types of evidence rather than social processes. “Consistency” is a two-part relation involving a theory component and an evidence component where the theory leads in some manner to the evidence. But what then is the role of social processes in constructing this knowledge?

Of course, millions of microsocial interactions occurred in the 15-year history of work on TRF from the early postulation of hypothalamic “factors” by Harris to Guillemin and Schally's breakthroughs of 1969. What we need to ask, though, is whether the theory and evidence at different stages were dependent on social processes? For example, if we examine Table 3 which deals with early clues that releasing factors are peptides, the work of Vincent du Vigneaud on vasopressin and oxytocin, both peptides from the pituitary, was an important influence on ideas regarding the structure of releasing factors, albeit through a formal rather than an informal communication channel. Similarly, Harris's isolation of melanophore stimulating peptide from the pituitary was formally communicated through the literature. More informally, ideas from embryology that glands developing from the ectoderm synthesize peptides were communicated to Guillemin through a lecture given by David Thompson (Guillemin and Lemke, 2013). Because the hypothalamus and the pituitary develop from the ectoderm, it seemed logical that releasing factors would follow that pattern.

Turning to Table 4 on TRF as a simple peptide, two cases of social interaction stand out. Guillemin's collaboration with chemist Darrell Ward on amino acid analysis which led to the glu-his-pro tripeptide, and Schally's collaboration with colleagues at Merck leading to the synthesis of all six permutations of the three amino acids and the finding of biological inactivity which contributed to the disconfirmation of the simple peptide model. In the first case, Guillemin was able to make the connection with TRF theory while Ward was not. In the second case, Schally performed the TRF assay and could make the disconfirming inference that none of the permutations led to activity while the Merck researchers were merely suppliers. Thus, knowledge was gained by only one party to the collaboration and was asymmetric.

Prominent social interactions for TRF as a protected peptide (Tables 5, 6) were the Roger Burgus collaboration with Guillemin which led to a revival of the three amino acid hypothesis and the hypothesis that the N-terminal was not a “free” NH_2_ group but was somehow protected, an idea that was first broached at the meeting of the endocrinology study section at Tucson. Guillemin's call to an acquaintance at Hoffmann-La-Roche in Switzerland to see if they could provide synthetic tripeptides paralleled Schally's earlier communication with Merck. Hoffmann-La-Roche also provided Burgus with the key suggestion that acetylation could have caused a pyro ring at the N-terminal to form, and as well as synthesizing the pure materials that led to pyro as the active form. It is doubtful that the Hoffmann-La-Roche researchers realized the theoretical implications of their information. Burgus, as Guillemin's chemist, would have realized the implications of these findings for TRF before Guillemin did, but, of course, would have shared them with Guillemin as the lab head. It was known that Guillemin was not actively involved at the lab bench during this period. Burgus' report that he was elated at finding only one of the six acetylated tripeptides was active shows that he realized its implications for TRF structure.

For Burgus and Guillemin, support for the amide form (Table 7) appears to have come from an analogy to other biologically active polypeptides which a knowledge of earlier literature could have revealed. Meanwhile, Schally's team had expanded to include a collaboration with chemists Folkers and Enzmann at Austin. Schally provided them with purified TRF and synthetic peptides from Merck. Enzmann then accidentally synthesized the amide form, sending the material back to Schally's lab for activity testing. Enzmann filled in a missing piece of the puzzle, that amidation could create a pyro ring, by searching the chemical literature. Enzmann working in Austin was clearly not under supervision by Schally in New Orleans but depended on Schally's group to test for TRF activity. Thus, the realization that amidation was somehow related to TRF was first achieved in New Orleans when Schally called Austin to say “We've got it.” A criticism by Schally that the peptide was not protected at the N-terminal was solved by Enzmann's literature search which helped cement the connection between TRF's amide form and its TRF activity. This was realized at Austin before it was communicated to New Orleans.

Social process such as reading prior literature, formal presentations, the sharing of materials, and direct communication and collaboration with colleagues facilitated “fact construction” and knowledge formation, providing at times important pieces of theory or evidence. However, like relevance judgments in linguistics (Wilson and Sperber, 2008), confirmation is driven by the two-part relation of theory to evidence, and the consistency between the two. In most of the cases described above only one side of the social interaction was in possession of both pieces of the puzzle and was able to see the consistency of theory and evidence. For example, when a published document provides an important clue, only the reader can see the connection. In a collaboration where one participant is providing synthesis or analytical services, it is only the recipient who will be able to see the connection. When Enzmann in Austin used amidation to create new compounds, he could not know what Schally knew after performing the assay. Again, the information was asymmetrical. In none of the instances of social interaction examined here would the evidence provided be confirming in and of itself in the absence of theory. The judgment of consistency that leads to confirmation can only come about by an individual who is in possession of both relevant pieces of theory and evidence. Of course, this consistency can be communicated to other members of the collaboration and eventually to the wider community. But each individual in the community needs to see the fit of theory and evidence to realize the confirmation. Clearly this is necessary before consensus can be reached. The puzzle analogy is appropriate here: whether two puzzle pieces fit together can only be perceived by participants in possession of both pieces simultaneously.

## 6. Discussion

It is clear from our Bayesian analysis that what Latour and Woolgar have called the construction of a “fact” is simply the most probable model given the evidence among the models that were proposed, that is, the model with the highest posterior probability. At the heart of the Bayesian computation are the conditional probabilities or likelihoods P(E|T) or P(E|~T) which express how well the theory T or ~T (not T) fit the evidence E. We need only assign these conditionals approximately taking the points of views of the participants. P(E|T) is equivalent to the conditional expression if T then E (Tweney and Yachanin, 1985) where E can speak either for or against T or ~T. Disconfirmation occurs if the alternative theories are more favored by evidence than the theory being tested, which occurred when the simple peptide model was disconfirmed, and likewise confirmation occurs only if the evidence disfavors the alternatives relative to the theory under scrutiny.

One aspect of this process we have not examined is its dependence on the perspective of the participant. For example, we have assumed that the Guillemin and Schally groups do not have different perspectives on what theories are at stake and what evidence is relevant and the strength of that evidence. The reason for this is that each group kept close tabs on the other, read each other's papers, attended the same meetings and were to some degree in direct communication. Thus, their hypotheses and relevant evidence were not significantly different, in fact it has been commented that their paths were remarkably parallel (Wade, 1981, p. 160). For example, we have combined the mass spectroscopy evidence from Guillemin's team and chromatographic evidence from Schally's team in Table 7 to compute the posterior for the amide form of TRF. Guillemin's claim of priority was based on his belief that mass spectroscopy evidence was stronger than chromatographic evidence, but changing the probabilities of those conditionals would not create a significant difference between the groups if their posterior had been computed separately.

However, the perspective of other players could have been quite different. For example, we have not examined the views of McCann at the University of Pennsylvania or Harris in England. Thus, we cannot claim that these individuals would have shared the same degree of certainty on the structure of TRF, just as Watson and Crick's model of DNA was not shared by Linus Pauling (Small, 2023). How a group or community of scientists arrives at a consensus on what theory is correct or evidence is most relevant is beyond the scope of this analysis, but a topic worthy of study given that consensus among experts is often used to justify scientific findings to the public (Cole, 1992; Oreskes, 2019).

Another question is how theories or models are formulated and how evidence is acquired. Evidence is usually experimental or observational but can also be more indirect and theory laden. For example, in Table 3 a theoretical generalization about embryological development is used to support the peptide nature of releasing factors. Analogies to other systems (e.g., embryonic development) are theoretical in the sense that they assume that the different systems will operate on the same principles. Of the 23 lines of evidence cited in our analyses, about 80% were experimental and 20% were analogies. Analogies played a role in the latter stages of the TRF search in the assessment of the acetylated and amide forms of TRF by appeal to the structure of other biological molecules. However, analogies can be misleading and require correction, for example, when Burgus thought TRF had the acetylated form, only to learn later that acetylation created a pyro ring.

The acquisition of evidence seems more a matter of chance and accident than plan. For example, Burgus' ability to perform mass spectroscopy on TRF was governed by his ability to make the molecule sufficiently volatile which he did not achieve until the fall of 1969 after earlier failures. Similarly, his finding that TRF was 80% glu-his-pro just weeks before the Tucson meeting, propelled the field to a new stage. Another example of good timing was when Enzmann learned from the literature that amidation could form a pyro ring on glu-his-pro-amide, and thus provided an explanation for its biological activity. The timing of this realization had a direct effect on when the pyro-glu-his-pro-amide model was confirmed. Thus, the most striking feature of the accumulation of evidence is its stochastic nature which is consistent with Latour and Woolgar's Brownian motion analogy. This argues that the pace of acquiring relevant evidence, and hence discovery, depends on access to as wide a network of information as possible from researchers who are fully informed on its theoretical implications.

It was noted earlier that Latour and Woolgar made frequent allusions to concepts from information theory such as probability and entropy, the former increasing and the latter decreasing as the “fact” is constructed. But how are these concepts related? Information theory as described in Brillouin's book which they cite (Brillouin, 1962) provides an answer which is closely related to Bayesian theory. The standard definition of entropy H, as introduced by Shannon, is H = -Σ_i_P(T_i_)Log_2_P(T_i_) where P is the probability of an event, the log is base 2, and the summation is over all possible states of the system (Cover and Thomas, 2006, p. 16). In the case where there are only two states, the theory T and negation of the theory ~T, the maximum entropy occurs when P(T) = 0.5 and P(~T) = 0.5, in which case H = 1 bit. This is the condition of greatest uncertainty. The lowest entropy (highest certainty) is when the probability of one theory greatly exceeds the other, that is, approaches 1.0 or zero, in which case entropy approaches zero.

Another result from information theory that has a bearing on Bayesian analysis is the concept of conditional entropy (Cover and Thomas, 2006, p. 17). We can express conditional entropy in terms of the conditional probabilities of Bayes' theorem as H = -Σ_T_Σ_E_P(T)P(E|T)Log_2_P(E|T) where the double summation is over T, ~T, E and ~E. This expression has a maximum value of 1.0, maximum uncertainty, when P(E|T) and P(E|~T) = 0.5 in which case the theory is neither confirmed nor disconfirmed. If either P(E|T) or P(E|~T) is 0.5 then the entropy has the highest average value when the other conditional is at 0.5. Thus, a conditional probability of 0.5, designated as “neutral” in Table 1, is the state of highest average entropy or uncertainty regarding how well the theory accounts for the evidence. Using the concepts of entropy and conditional entropy from information theory provides a unified framework for setting priors and conditional probabilities.

At the beginning of the TRF story (Table 3) we set the prior probability of the peptide hypothesis P(T) to 0.5 to reflect its high uncertainty using the principle of indifference, making the alternative hypothesis P(~T) also 0.5. Hence, the entropy was a maximum of 1.0. In the mid-1960's when the simple peptide model was disconfirmed, the entropy fell from about 0.9 to 0.7 because disconfirmation is a more certain state than even odds. In 1969 the posterior of the final pyro-glu-his-pro-amide hypothesis was 0.99 giving it an entropy close to zero. This was a precipitous decline from the previous pyro-glu-his-pro model having an entropy of about 0.6. This confirms Latour and Woolgar's intuition that entropy was reduced when “fact” status was achieved. Simply stated, entropy declines as the posterior approaches 1.0 (total certainty) or approaches zero (impossibility). This means that Maxwell's demon operates in the lab, to use Latour and Woolgar's metaphor, by accepting hypotheses whose posteriors exceed their priors and rejecting hypotheses whose posteriors are below their priors which results in both cases reducing the entropy. The demon thus operates a Bayesian trap door. We could also speculate that the microsocial interactions occurring in the lab where the modalities of statements are added or dropped reflect the day-to-day changes in the posteriors of the hypotheses as evidence comes in real time either increasing or decreasing the entropy in the lab.

## 7. Conclusions

In Bayesian terms scientific knowledge results from an interplay of theories and evidence and is driven by the accumulation of evidence consistent or inconsistent with a theory or hypothesis. Knowledge, however, is never immune to revision. A theory is at best more probable than some alternative hypothesis. The fundamental atom of knowledge is the conditional probability that a theory can predict or explain some form of evidence expressed as a likelihood P(E|T), the probability of evidence E being consistent with theory T, where E can be an empirical observation or a theoretical claim.

Social processes in science, such as formal or informal communication, act as a suggestion box for potential changes in theory or evidence, and either facilitate or retard the acquisition of theoretical ideas and new forms of relevant evidence. Most of the suggestions communicated prove to be dead ends but a small fraction turn out to be decisive for advancing confirmation or disconfirmation. However, the fit between theory and evidence can only be perceived by individuals in possession of both components because confirmation is driven by two-place conditionals. In many cases of social interaction in the TRF case, knowledge by the parties involved was asymmetric, with one party knowing both the theory and evidence and able to assess the fit, and the other party knowing only one side of the story. Controlling the flow of information—who knows what—is important in the competition for priority but can impede confirmation. Thus, social processes can accelerate or decelerate new knowledge or “fact” formation but do not do the work of confirmation.

The Bayesian account of the TRF story arrives at the same end point as Latour and Woolgar's constructivist account, namely highly probable knowledge. But the two accounts arrive at that end point in quite different ways and are ultimately incompatible. The motive force for the constructivist approach involves microsocial processes and convincing colleagues, while the Bayesian approach is driven by the consistency of theory and evidence as judged by each participant. The actors may agree with one another and achieve a consensus, but it is a consensus of individuals who have each separately perceived the theory-evidence fit. In the Bayesian approach the judgement of consistency must precede consensus and the convincing of colleagues.

While we have used the terms model, theory, and hypothesis interchangeably, it is not difficult to see how the Bayesian methods could be extended to other types of discourse, for example, scientific methods, instruments, and even technologies. A method or technology, like a scientific theory or model, is directed toward an end. One such end is the structure of a hormone, and another is a method to assay a hormone or to separate a complex mixture. A method can be compared with competing methods or techniques according to their advantages and disadvantages using the same types of conditional probabilities we have used to assess theories and models. These Bayesian extensions into technology remain to be explored.

## Data availability statement

The raw data supporting the conclusions of this article will be made available by the authors, without undue reservation.

## Author contributions

HS conceived the study, collected and analyzed the data, and wrote the paper.
